# A morphological and molecular study of phlebotomine sand flies of Taiwan reveals the record of six species

**DOI:** 10.1186/s13071-025-07017-1

**Published:** 2025-10-09

**Authors:** Han-Hsuan Chung, Cheng-Hui Tsai, Hwa-Jen Teng, Shiu‐Ling Chen

**Affiliations:** Center for Diagnostics and Vaccine Development, Centers for Disease Control, Ministry of Health and Welfare, Taipei, 115210 Taiwan

**Keywords:** Phlebotomine sand fly, Leishmaniasis, Vector, Transmission risk, *COI*, *18S rDNA*, *Cytb*, Taiwan

## Abstract

**Background:**

Phlebotomine sand flies, the key vectors in the transmission of *Leishmania* parasites, pose a global health challenge. Although leishmaniasis has been reported in the indigenous Taiwanese population, the last sand fly survey, based on morphological features, was conducted over two decades ago. Thus, updated information on the phlebotomine sand fly fauna and disease transmission risk in Taiwan is required. In this study, a nationwide survey was conducted using molecular methods to ascertain the current sand fly status and disease transmission risk in Taiwan.

**Methods:**

A total of 1292 sand flies were collected in a nationwide survey conducted in 2017–2018. Species were identified based on their 18S ribosomal DNA (*18S rDNA*), cytochrome c oxidase subunit I (*COI*), and cytochrome b (*Cytb*) using the phylogenetic tree and intra- and interspecific divergence analysis. The relative abundance, richness, diversity, and evenness of sand fly species were also calculated.

**Results:**

Phylogenetic analysis showed six independent clades, including *Phlebotomus kiangsuensis* (0.1%), *Sergentomyia iyengari* (93.7%), *Sergentomyia barraudi* (3.8%), *Sergentomyia squamipleuris* (1.6%), and two species described for the first time (0.9%). Species divergence analysis supported the phylogenetic results. The richness and abundance of sand flies were higher in eastern Taiwan than in western regions. Blood-source analysis showed an interaction between *Se. iyengari* and humans. In addition, no *Leishmania* spp. DNA was detected in any specimen, which suggests a low transmission risk for *Leishmania* spp. in Taiwan.

**Conclusions:**

These findings provide valuable knowledge on the current fauna of phlebotomine sand flies, which is beneficial for assessing disease risk and managing vector control in Taiwan.

**Graphical abstract:**

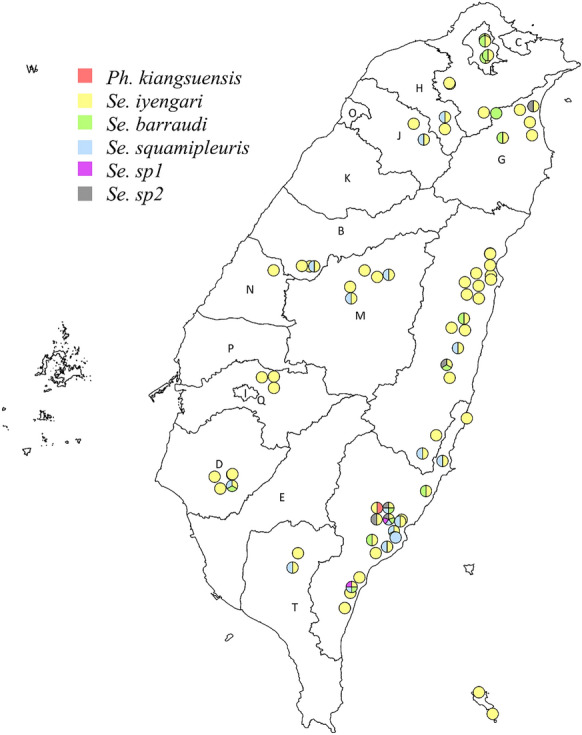

**Supplementary Information:**

The online version contains supplementary material available at 10.1186/s13071-025-07017-1.

## Background

Leishmaniasis, a parasitic disease caused by a protozoan of the *Leishmania* spp., occurs widely in tropical and subtropical regions [1]. Leishmaniasis is endemic to more than 90 countries, especially in the Middle East, Africa, Asia, and Central and South America [2]. It is estimated that approximately 700,000 to 1,000,000 new cases occur worldwide annually [3]. Leishmaniasis is categorized into visceral, cutaneous, and mucocutaneous forms based on its clinical manifestations [2]. In Taiwan, leishmaniasis can be traced back to the end of World War II in the 1950s, when five evacuated soldiers were diagnosed with either visceral leishmaniasis or post-kala-azar dermal leishmaniasis [4]. However, it is not considered an endemic disease in Taiwan, as only six cases have been reported over the past six decades. The first autochthonous cases of cutaneous-subcutaneous leishmaniasis were reported in the 1960s in two native-born residents. However, the arthropod and animal examinations conducted in the vicinity of these two cases could not detect the presence of the parasite [5]. In 2008, Wang et al. reported that indigenous *Leishmania tropica* caused cutaneous leishmaniasis in an individual [6]. The last reported cases of leishmaniasis in Taiwan occurred between 2005 and 2007; these were three indigenous cutaneous cases in southern Taiwan wherein *L. tropica* was detected in only one individual [7]. These six indigenous cases suggest the presence of the disease vector responsible for the transmission of leishmaniasis in Taiwan. In addition, imported leishmaniasis cases from endemic countries have also been documented, which underlines the global extent of the parasite [8, 9]. Furthermore, infection by *Streptococcus pyogenes* after sand fly bites has also been reported [10]. Therefore, understanding the species and distribution of sand flies in Taiwan will be beneficial for risk assessment and prevention of leishmaniasis.

Phlebotomine sand flies are a subfamily of the family Psychodidae. There are 40 genera of sand flies in the world according to an updated checklist [11]. Three genera that have been classified within the Phlebotominae subfamily, include *Phlebotomus*, *Lutzomyia*, and *Sergentomyia*. Of these, the first two are believed to be important vectors in the transmission of leishmaniasis, bartonellosis, and sand fly fever in the Old and New World [1]. In Taiwan, four species of phlebotomine sand flies have been described, namely *Phlebotomus kiangsuensis*, *Sergentomyia iyengari*, *Sergentomyia barraudi*, and *Sergentomyia squamipleuris*. Among them, *Ph. kiangsuensis* is believed to be a suspected vector of *Leishmania* spp. due to its tendency to feed on human blood [12, 13]. However, no new information related to *Ph. kiangsuensis* has been reported since its first record in 1970 in Taiwan. In contrast to the rarely found *Phlebotomus* spp., Lin et al. found that *Se. iyengari* was the predominant sand fly species in Taiwan, followed by *Se. squamipleuris* and *Se. barraudi* [14]. They also documented three additional species of sand flies with distinct morphological characteristics [14]. In a surveillance conducted in 2005 around the residence of an indigenous case to elucidate the transmission route, all collected sand fly specimens belonged to the *Se. iyengari* [6, 15]. Until now, all the sand fly species reported in Taiwan have been identified based on their morphological characteristics [12, 14]. With the advancements in biotechnology, molecular identification of sand flies is helpful in identifying morphologically similar species, especially in small-sized sand flies. The sequences of the partial 18S ribosomal DNA (*18S rDNA*), cytochrome c oxidase subunit I (*COI*), and cytochrome b (*Cytb*), which are beneficial for tracing the origin of sand fly migration and distinguishing species, including sibling species differentiation, have been widely utilized in sand fly studies [16–19].

Contrastingly, although *Ph. kiangsuensis* is a suspected vector for leishmaniasis in Taiwan, it is believed that the *Sergentomyia* spp. do not act as vectors for human leishmaniasis because they preferentially feed on cold-blooded vertebrates and act as vectors for reptilian leishmaniasis [20]. However, the medical importance of *Sergentomyia* spp. as potential vectors for *Leishmania* spp. transmission has been highlighted in some studies [21–23]. For example, the detection of the DNA of *Leishmania* spp. in *Se. iyengari*, *Se. gemmea*, and *Se. khawi* provides evidence supporting their potential role as vectors for leishmaniasis. Therefore, further information is required on the status of *Leishmania* spp. in different sand fly species in Taiwan.

In Taiwan, the current data on phlebotomine fauna is inadequate, and relevant information is needed to identify the possible transmission route and evaluate the risk of *Leishmania* transmission. In this study, we conducted a nationwide surveillance of phlebotomine sand flies in Taiwan. The analysis of blood-meal source and the detection of *Leishmania* spp. among field sand flies was also carried out to determine their potential role in disease transmission.

## Methods

### Collection of phlebotomine sand flies

Sand flies were collected in western and eastern Taiwan in 2017 and 2018, respectively. We set up a black-light suction trap (Model 1312; John W. Hock Company, FL, USA), accompanied by dry ice, before sunset, to attract sand flies overnight. The inlet of the light trap was set at a height of less than 50 cm above ground level. The samples were harvested the next morning (Additional file 1: Fig. S1) [14, 24]. Sampling sites were preferentially selected in rural areas where the land was covered with moist soil and decaying leaves, especially in shaded areas. Soil beds along clean streams were also chosen for sampling sites (Additional file 2: Table S1). We also selected recreational areas to investigate the ecology of sand flies for risk assessment of disease transmission. At least three sites were chosen for sampling in each county or city, including the residential areas of previously confirmed cases of leishmaniasis. Two offshore islands belonging to Taitung, the Green and Orchid Islands, were also included. One trap was deposited in each sampling site. The relative abundance, richness, diversity [Simpson’s dominance index (1-D)], and evenness [Shannon–Wiener index *H*’/ln(richness)] of sand fly species were calculated according to a previous study [25]. The Global Positioning System (GPS) coordinates of all collection sites were recorded. Samples were kept on dry ice and brought back to the laboratory for immediate identification or stored at −80 °C in a refrigerator.

### QGIS (Quantum Geographic Information System) mapping

We used QGIS software (version 3.32, http://www.qgis.org/en/site/) to demonstrate the sampling sites and species distribution of the sand flies. In brief, the TWD97 Taiwan township map provided by the National Development Council Government Website Open Information (https://data.gov.tw/dataset/7442) was utilized as the data layer. The GPS-coordinated data (Additional file 2: Table S1) were plotted on the map using the EPSG:3824 system.

### Morphological identification

Phlebotomine sand flies were identified using a dissecting microscope based on morphological keys described previously [12]. Pretreatment of sand fly samples was conducted as described in a previous study with a few modifications [14]. In brief, the head of each sand fly was removed and immersed in 10% potassium hydroxide overnight, then washed twice with deionized water. The transparent sample was then soaked in 0.1 N sodium acetate for 30 min and washed twice with deionized water. Samples were stained with 0.5% carbol fuchsin (ref. no. HT8108; Sigma-Aldrich, MA, USA) for 30 s. After staining, the cibarium and pharynx were dissected from the head; the number and size of the cibarial teeth, shape of pharyngeal armature, and sclerotized area were examined under the dissecting microscope for species identification according to the morphological key [12].

### Molecular identification of sand flies

All male and female sand fly genomic DNA was extracted using the QIAamp DNA Mini Kit (cat. no. 51306; QIAGEN, Germany) from the abdomen (the head of the specimen was used for morphological identification) or the whole sand fly. The *18S rDNA*, *COI*, and *Cytb* were amplified by polymerase chain reaction (PCR) using a specific primer pair (Ph18S F: 5′-TAGTGAAACCGCAAAAGGCTCAG-3′ and Ph18S R: 5′-CTCGGATGTGAGTCCTGTATTGT-3′; COI F: 5′-GGTCAACAAATCATAAAGATATTGG-3′ and COI R: 5′-TAAACTTCAGGGTGACCAAAAAATCA-3′; Cytb F N1N: 5′-CAYATTCAACCWGAATGATA-3′ and Cytb R C3B: 5′-GGTAYWTTGCCTCGAWTTCGWTATGA-3′) [26–28]. The PCR cycling conditions were as follows: initial denaturation at 94 °C for 2 min, followed by 38 cycles of denaturing at 94 °C for 30 s, annealing at 55 °C for 30 s, and extension at 72 °C for 60 s. The final extension was carried out at 72 °C for 4 min. The PCR products were sequenced and aligned to determine the nucleotide differences among species using BioEdit built-in ClustalW version 7.2.5 software (https://bioedit.software.informer.com/). The Basic Local Alignment Search Tool nucleotide (BLASTn) suite in the National Center for Biotechnology Information (https://blast.ncbi.nlm.nih.gov/Blast.cgi) was used to identify the sand fly sequences (randomly selected one sequence of each species) obtained in this study.

### Phylogenetic analysis and sequence divergence

One representative specimen was selected randomly for analysis when multiple sequence specimens shared the same haplotype. The *18S rDNA*, *COI*, and *Cytb* sequences of relevant species were also retrieved from the NCBI GenBank or the BOLD Systems database for comparison. Phylogenetic analysis and intra- and interspecific divergence calculation were performed using MEGA11 [Molecular Evolutionary Genetics Analysis version 11] software [29]. In summary, *18S rDNA*, *COI*, and *Cytb* sequences were trimmed for alignment using MUSCLE. The phylogenetic trees were constructed using maximum likelihood (ML) and neighbor-joining (NJ) methods based on the model with the lowest Bayesian information criterion. The pairwise deletion was used to treat missing data. Bootstrap values were estimated using 1000 replicates.

### Blood meal and *Leishmania* spp. DNA detection

Genomic DNA extracted from the abdomen or whole female sand fly (*n* = 898) was subjected to a PCR-based assay to check the blood source by specific primer sets for avian (Avian-3F 5′-GACTGTGAYAAAATYCCMTTCCA-3′; Avian-8R 5′-GYCTTCAITYTTTGGYTTACAAGAC-3′) and mammalian (Mammalian-1F 5′-TGAYATGAAAAAYCATCGTTG-3′; Mammalian-2R 5′-TGTAGTTRTCWGGGTCKCCTA-3′) *Cytb* gene [30]. The PCR cycling conditions for the mammalian *Cytb* gene were as follows: initial denaturation at 94 °C for 2 min, followed by 35 cycles of denaturing at 94 °C for 30 s, annealing at 55 °C for 30 s, and extension at 72 °C for 45 s. The final extension was conducted at 72 °C for 5 min. For avian *Cytb*, initial denaturation was carried out at 94 °C for 2 min, followed by 35 cycles of denaturation at 94 °C for 30 s, annealing at 52 °C for 30 s, and extension at 72 °C for 60 s. The final extension was conducted at 72 °C for 5 min. The genomic DNA of chicken and mouse blood was used as the positive control. The amplicons were sent out for direct sequencing (Genomics, New Taipei, Taiwan) by PCR primers. To detect *Leishmania* parasites, specific primers for *Leishmania* ribosomal internal transcribed spacer (ITS) (LITSR: 5′-CTGGATCATTTTCCGATG-3′; L5.8S: 5′-TGATACCACTTATCGCACTT-3′) were used and PCR was performed under the following conditions: initial denaturation at 94 °C for 2 min, followed by 35 cycles of denaturation at 94 °C for 30 s, annealing at 53 °C for 30 s, and extension at 72 °C for 60 s. The final extension was conducted at 72 °C for 5 min [31]. The *Leishmania major ITS1* gene cloned into the TA vector (cat. no. K202020; Invitrogen, CA, USA) was used as the positive control.

## Results

**Fig. 1:**
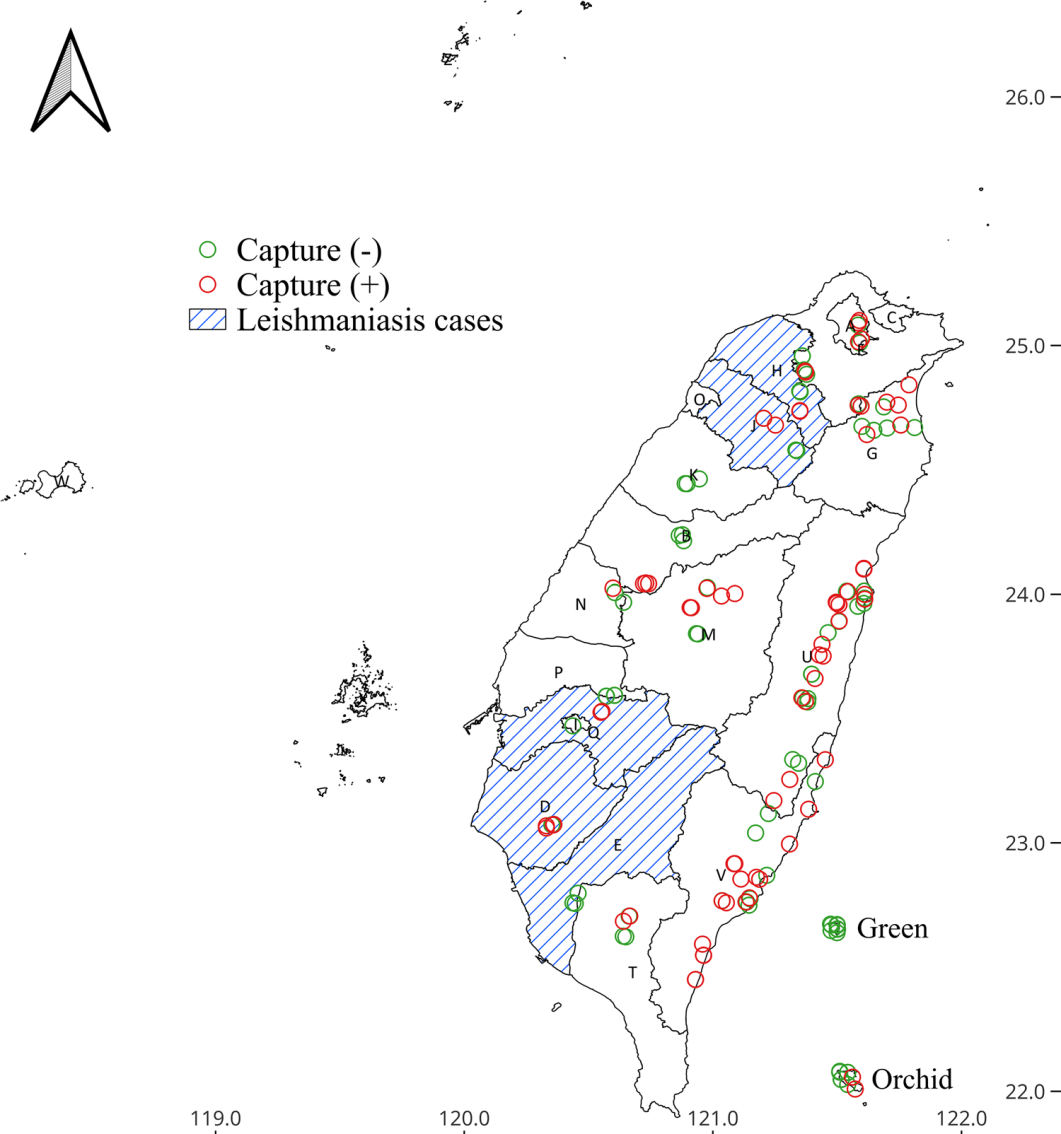
Map of sand fly collection sites in Taiwan. Circles represent collection localities: green circles indicate the sites where sand fly capture failed; red circles indicate sites with successful sand fly capture. The blue-shaded area represents the districts with reported leishmaniasis cases. City and county abbreviations are labeled on the map. Administrative districts in western Taiwan include Taipei City (A), New Taipei City (F), Taoyuan City (H), Hsinchu County (J), Hsinchu City (O), Miaoli County (K), Taichung City (B), Nantou County (M), Changhua County (N), Yunlin County (P), Chiayi County (Q), Chiayi City (I), Tainan City (D), Kaohsiung City (E), and Pingtung County (T). Administrative districts in eastern Taiwan are Ilan County (G), Hualien County (U), and Taitung County (V). The map was created by QGIS 3.32.2 (https://qgis.org)

**Fig. 2:**
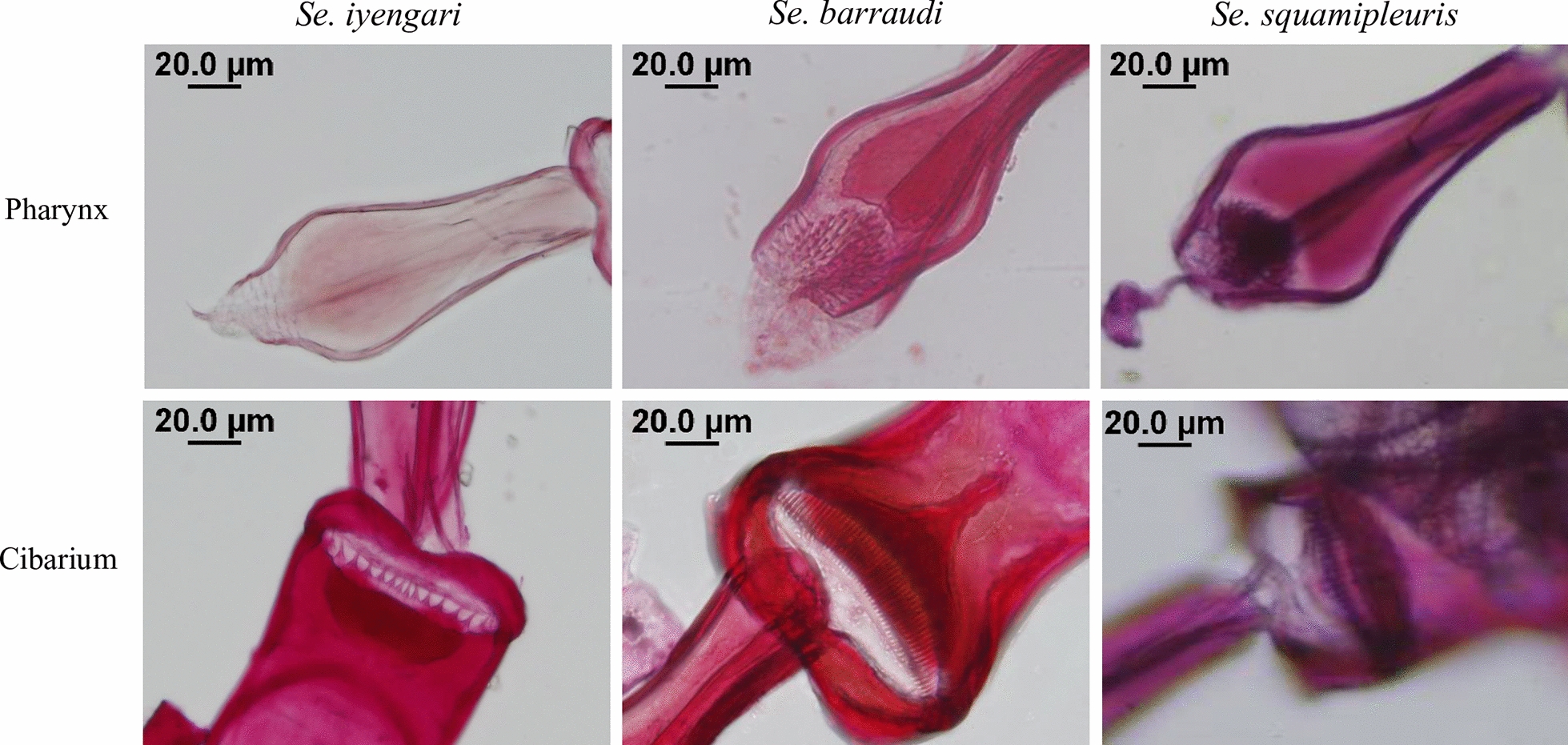
Morphological features of the cibarial and pharyngeal armature of sand flies. Representative morphological features of the pharyngeal (upper panel) and cibarial (lower panel) structures from *Se. iyengari*, *Se. barraudi*, and *Se. squamipleuris*, respectively. Scale bar: 20 μm

Between 2017 and 2018, a survey was conducted across 168 different locations in 16 cities in Taiwan (Additional file 2: Table S1), including the cities where the indigenous cases were sampled. Of these locations, 64 were in western Taiwan and 104 were in eastern Taiwan (Fig. 1). Sand flies were found at 76 sites (Fig. 1). The sand flies collected from western Taiwan in 2017 were randomly selected for morphological identification, revealing three previously reported species: *Se. iyengari*, *Se. barraudi,* and *Se. squamipleuris* (Fig. 2). *Se. iyengari* exhibits a wide sclerotized area with a slightly concave middle. The cibarium bears 14–19 uniform posterior teeth, with the central four positioned relatively close together. The pharyngeal armature contains short, fragment-shaped denticles arranged in a parallel and interlaced pattern. *Se. barraudi* features a bell-shaped sclerotized area with distinctly separated ends. The cibarium contains 40–48 small posterior teeth. The pharyngeal armature is equipped with conspicuous teeth. *Se. squamipleuris* has 30–35 posterior teeth in the cibarium. The pharynx is posteriorly expanded and densely armed with prominent teeth.

**Fig. 3:**
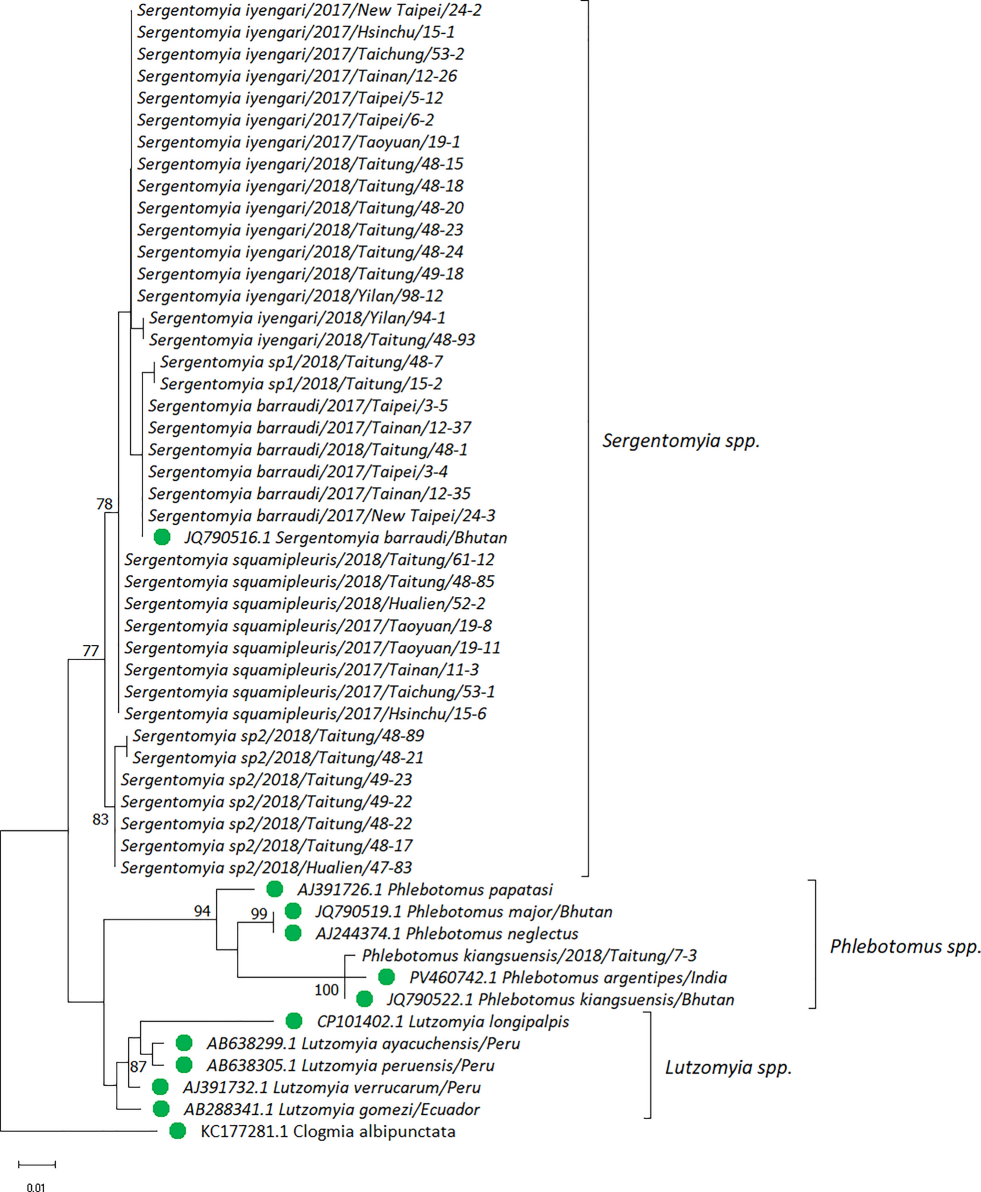
Phylogenetic analysis of phlebotomine sand flies based on 18S ribosomal DNA (*18S rDNA*) sequences. The evolutionary tree was constructed using the maximum likelihood method. Evolutionary distances were computed using the Tamura 3-parameter model. This analysis involved 52 nucleotide sequences with a total of 347 positions in the final dataset. The bootstrap value (1000 replicates) at which the associated taxa clustered together is shown at nodes when this value is above 70. The green circle indicates the reference sequence retrieved from the NCBI or BOLD Systems database. *Clogmia albipunctata* was set as the outgroup. Evolutionary analyses were performed in MEGA11

**Fig. 4:**
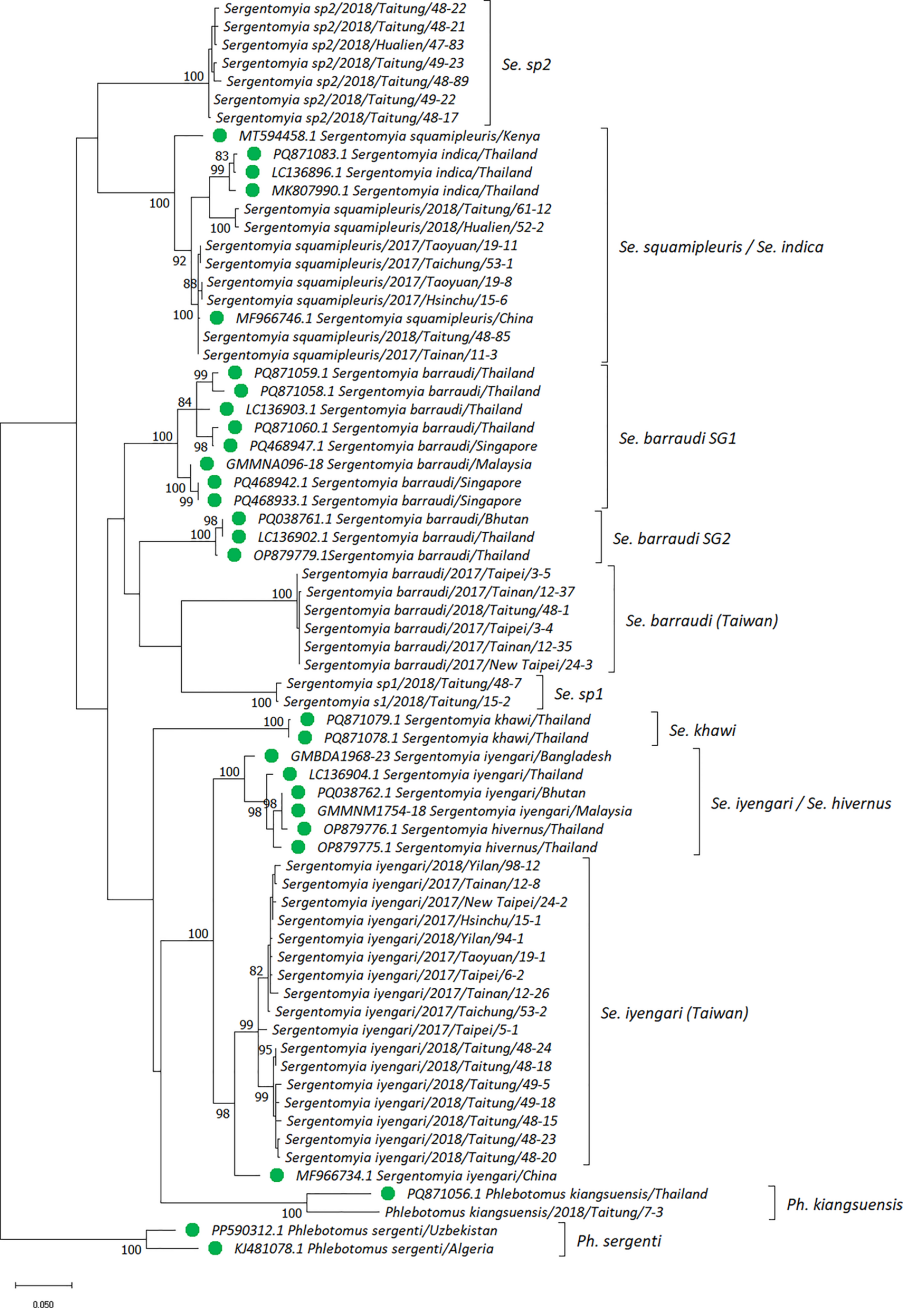
Phylogenetic analysis of phlebotomine sand flies based on cytochrome c oxidase subunit I (*COI*) sequences. The evolutionary tree was constructed using the maximum likelihood method and general time-reversible model. This analysis involved 69 nucleotide sequences with a total of 586 positions in the final dataset. The bootstrap value (1000 replicates) at which the associated taxa clustered together is shown at nodes when this value is above 70. The green circle indicates the reference sequence retrieved from the NCBI or BOLD Systems database. *Ph. sergenti* was set as the outgroup. Evolutionary analyses were performed in MEGA11

**Fig. 5:**
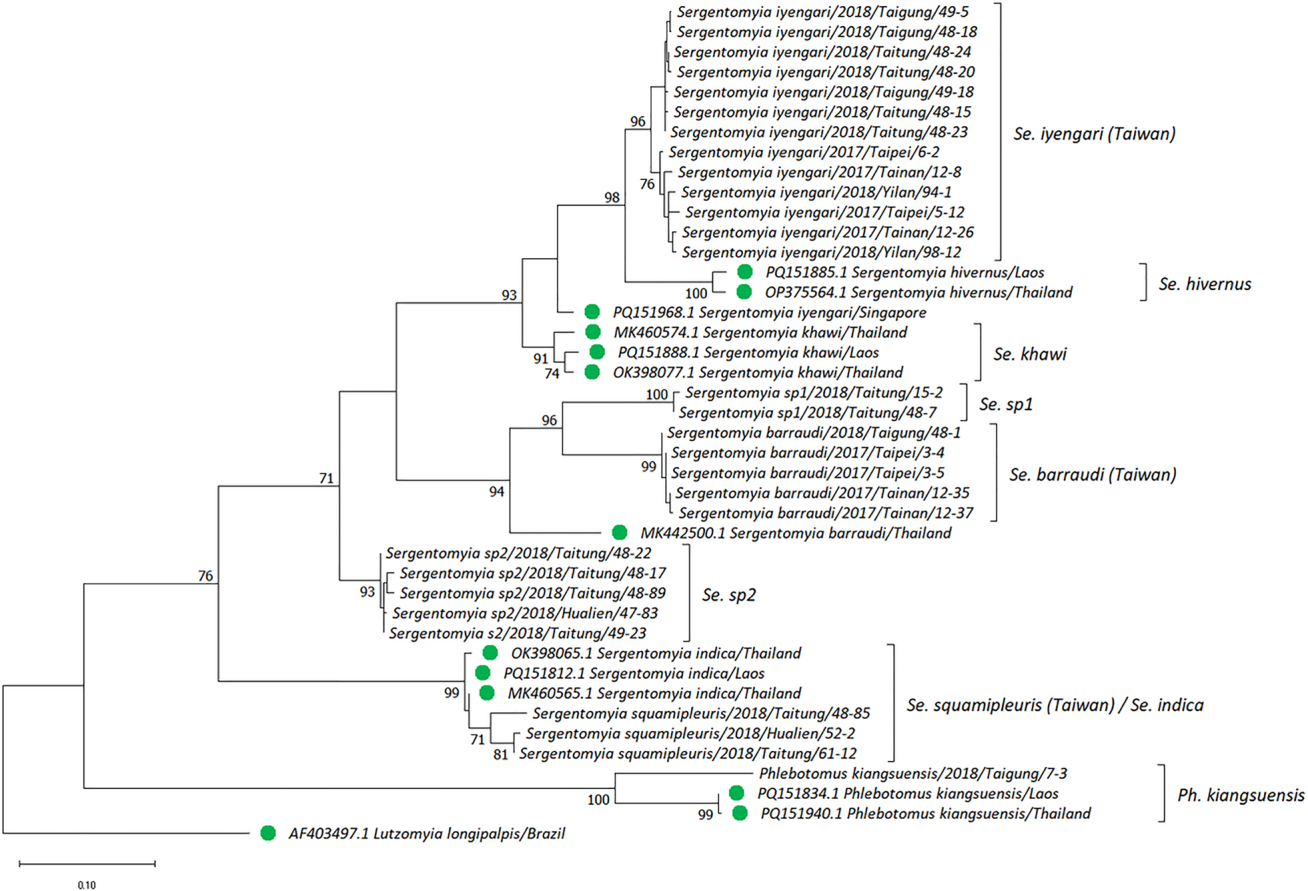
Phylogenetic analysis of phlebotomine sand flies based on cytochrome b (*Cytb*) sequences. The evolutionary tree was constructed using the maximum likelihood method and the Tamura 3-parameter model. This analysis involved 42 nucleotide sequences with a total of 441 positions in the final dataset. The bootstrap value (1000 replicates) at which the associated taxa clustered together is shown at nodes when this value is above 70. The green circle indicates the reference sequence retrieved from the NCBI or BOLD Systems database. *Lutzomyia longipalpis* was set as the outgroup. Evolutionary analyses were performed in MEGA11

To confirm the species, all the sand flies in this study were subjected to molecular sequencing of their *18S rDNA*, *COI*, and *Cytb*. The representative sequences were subsequently used to construct the phylogenetic tree (these sequences were deposited in NCBI GenBank, accession numbers PQ819521–PQ819560, PQ819580–PQ819620, and PV664330–PV664358 for *18S rDNA*, *COI*, and *Cytb*, respectively). The phylogenetic trees were further constructed for analysis. For *18S rDNA*, *Sergentomyia* spp., *Phlebotomus* spp., and *Lutzomyia* spp. formed three groups. It was surprising to observe that one sample collected in Taitung was identified as *Ph. kiangsuensis*, clustering with the same species collected in Bhutan and India. We also observed two subgroups of sand flies that belonged to *Sergentomyia* spp. (*Se.* sp1 and *Se.* sp2). Notably, the *Se.* sp2 samples comprised a monophyletic clade with a bootstrap value of 83 (Fig. 3 and Additional file 1: Fig. S2). To validate the results observed with *18S rDNA*, a phylogenetic tree was also constructed using the *COI* and *Cytb* genes. In *COI*, four strongly supported monophyletic clades, namely *Se. iyengari*, *Se. barraudi*, *Se.* sp1, and *Se.* sp2, were identified, with bootstrap values exceeding 99. However, the *COI* sequences of *Se. squamipleuris* collected from Taiwan, China, and Kenya formed a supergroup (bootstrap value = 99) with those of *Se. indica*. In addition, the clade of *Se. iyengari* from Taiwan was separated from those of the *Se. iyengari*/*Se. hivernus* from other Asian countries and *Se. khawi*. Moreover, the *Se. barraudi* from Taiwan was distinct from *Se. barraudi* samples from other countries of Asia, which also diverged into two distinct lineages (SG1 and SG2). Similarly, the *COI* sequence of *Ph. kiangsuensis* formed a distinct lineage, separate from the *Sergentomyia* (Fig. 4 and Additional file 1: Fig. S3). In *Cytb*, although all *Sergentomyia* formed a supergroup with the bootstrap value of 76, a tree topology similar to that constructed by *COI* was observed (Fig. 5 and Additional file 1: Fig. S4).

The BLAST results for *COI* and *Cytb* are shown in Table 1. *Ph. kiangsuensis* matched its nominal species from Thailand for both *COI* and *Cytb*, with the identity ranging from 90.8% to 91.6%. The *COI* sequences of *Se. squamipleuris* showed 100% identity to *Se. squamipleuris* from China; however, the *Cytb* showed 96.9% identity to *Se. indica* from Thailand. For *Se. iyengari*, the *COI* sequence showed 95.4% identity with *Se. iyengari* from China, while the *Cytb* sequence exhibited 93.9% identity with *Se. hivernus* from Laos, suggesting a close relationship. For *Se. barraudi*, *COI* and *Cytb* sequences matched *Se. insularis* and *Se. siamensis*, respectively, with identity lower than 91.7%, indicating a genetic gap. In contrast, *Se.* sp1 and *Se.* sp2 showed lower identities (ranging from 89.6% to 92.2%) to several known *Sergentomyia* sand flies, suggesting that they may represent distinct species not yet described in the current NCBI database.

**Table 1:** The *COI* and *Cytb* gene sequences obtained from sand flies collected in this study analyzed using BLASTn against the NCBI database

Present study	*COI* (NCBI)				*Cytb* (NCBI)			
Species^a^	Species	Country	Identity (%)	Accession no.	Species	Country	Identity (%)	Accession no.
*Se. iyengari* (48-18)	*Se. iyengari*	China	95.4	MF966734.1	*Se. hivernus*	Laos	93.9	PQ151885.1
*Se. barraudi* (48-1)	*Se. insularis*	India	89.2	HQ585365.1	*Se. siamensis*	Laos	91.7	PQ151928.1
*Se. squamipleuris* (48-85)	*Se. squamipleuris*	China	100.0	MF966747.1	*Se. indica*	Thailand	96.9	MK460565.1
*Se.* sp1 (48-7)	*Se. barraudi*	Thailand	89.6	OP879779.1	*Se. barraudi*	Thailand	90.0	MK442500.1
*Se.* sp2 (48-17)	*Se. perturbans*	Thailand	89.9	LC136897.1	*Se. phasukae*	Laos	92.2	PQ151911.1
*Ph. kiangsuensis* (7-3)	*Ph. kiangsuensis*	Thailand	91.6	PQ871056.1	*Ph. kiangsuensis*	Thailand	90.8	PQ151940.1

^a^The number in the parentheses indicates the site number and the specimen number

The intra- and interspecific divergence was calculated based on the *COI* (Table 2) and *Cytb* (Table 3) sequences. In *COI*, the intraspecific divergence ranged from 0.0012 to 0.0584, except for the *Ph. kiangsuensis* (0.1397). The interspecific divergence ranged from 0.0912 to 0.4949, except for the variations between *Se. squamipleuris* and *Se. indica* (0.0517), and *Se. iyengari* and *Se. hivernus* (0.0417). In *Cytb*, the intraspecific divergence ranged from 0.0033 to 0.0285, except for the *Ph. kiangsuensis* (0.0828). The interspecific divergence ranged from 0.0826 to 0.4133, except for the variations between *Se. squamipleuris* from Taiwan and *Se. indica* (0.0352). The interspecific divergences of *Se.* sp1 and *Se.* sp2 with other species were all greater than 0.1139, exceeding the intraspecific divergence observed within *Se.* sp1 (0.0047) and *Se.* sp2 (0.0071), respectively.

**Table 2:** The intra- and interspecific divergence between 67 *COI* sequences of phlebotomine sand flies

Species (*n*)	*Ph.* *kiangsuensis*	*Se. iyengari* (Taiwan)	*Se. barraudi* (Taiwan)	*Se.* *squamipleuris*	*Se.* sp1	*Se.* sp2	*Se.* *iyengari*	*Se.* *hivernus*	*Se. barraudi* SG1	*Se. barraudi* SG2	*Se. indica*	*Se.* *khawi*
*Ph. kiangsuensis* (2)	0.1397											
*Se. iyengari* (Taiwan) (17)	0.3433	0.0205										
*Se. barraudi* (Taiwan) (6)	0.3811	0.3736	0.0012									
*Se. squamipleuris* (10)	0.4949	0.3312	0.3150	0.0326								
*Se.* sp1 (2)	0.4563	0.3308	0.2648	0.3087	0.0072							
*Se.* sp2 (7)	0.3736	0.3675	0.2728	0.2276	0.2867	0.0087						
*Se. iyengari* (5)	0.3689	0.0912	0.3517	0.2974	0.3172	0.3128	0.0584					
*Se. hivernus* (2)	0.3738	0.1055	0.3497	0.3130	0.3251	0.3233	0.0417	0.0216				
*Se. barraudi* SG1 (3)	0.4053	0.2629	0.2349	0.2541	0.2640	0.2786	0.2399	0.2413	0.0394			
*Se. barraudi* SG2 (8)	0.3788	0.2611	0.2355	0.2436	0.1894	0.2635	0.2622	0.2623	0.1686	0.0047		
*Se. indica* (3)	0.4178	0.2969	0.2557	0.0517	0.2969	0.2106	0.3028	0.2902	0.2547	0.2488	0.0097	
*Se. khawi* (2)	0.4515	0.3143	0.2959	0.2964	0.3405	0.3113	0.2686	0.2710	0.2522	0.3594	0.3103	0.0017

**Table 3:** The intra- and interspecific divergence between 39 *Cytb* sequences of phlebotomine sand flies

Species (*n*)	*Ph.* *kiangsuensis*	*Se. iyengari* (Taiwan)	*Se. barraudi* (Taiwan)	*Se. squamipleuris* (Taiwan)	*Se.* sp1	*Se.* sp2	*Se.* *hivernus*	*Se.* *indica*	*Se.* *khawi*
*Ph. kiangsuensis* (3)	0.0828								
*Se. iyengari* (Taiwan) (13)	0.3448	0.0163							
*Se. barraudi* (Taiwan) (5)	0.3410	0.2130	0.0033						
*Se. squamipleuris* (Taiwan) (3)	0.3643	0.2444	0.2759	0.0285					
*Se.* sp1 (2)	0.4133	0.2475	0.1296	0.3578	0.0047				
*Se.* sp2 (5)	0.3073	0.1529	0.1493	0.2125	0.1837	0.0071			
*Se. hivernus* (2)	0.3670	0.0826	0.2197	0.2342	0.2437	0.1545	0.0176		
*Se. indica* (3)	0.3449	0.1955	0.2514	0.0352	0.3035	0.1866	0.2045	0.0048	
*Se. khawi* (3)	0.3239	0.0963	0.1713	0.2168	0.2087	0.1139	0.1097	0.1856	0.0226

**Fig. 6:**
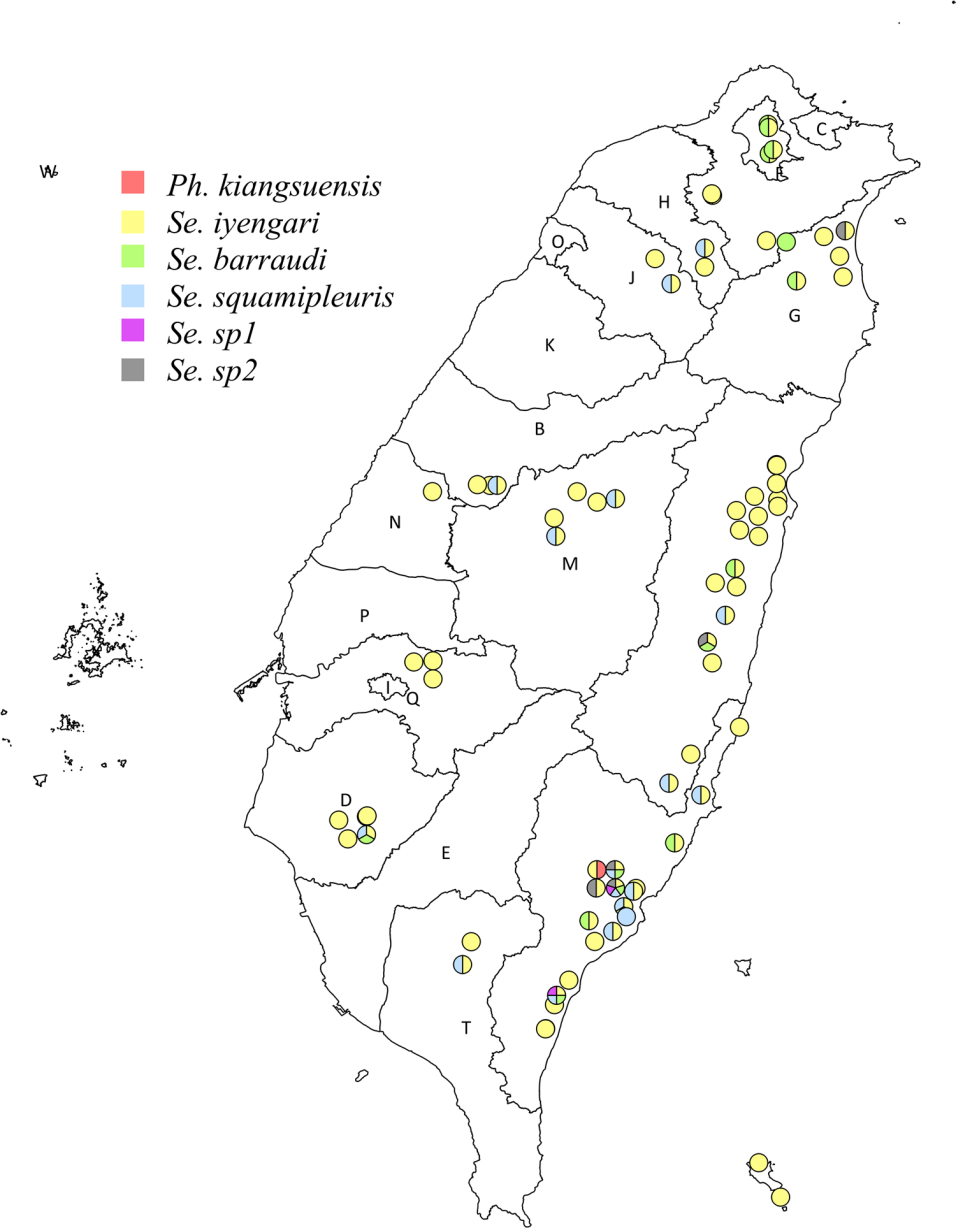
Species distribution of sand flies in Taiwan. Each pie chart represents a locality where sand flies were captured, showing the species composition at each site. The map was created using QGIS 3.32.2 (https://qgis.org)

According to the species identified by molecular methods, the distribution of sand flies in Taiwan is presented in Fig. 6 and Additional file 2: Table S1. *Se. iyengari* was widely distributed across the entire island of Taiwan, including Orchid Island. Among the sand flies captured in various localities, 97.4% (74/76) were *Se. iyengari*, 22.4% (17/76) were *Se. squamipleuris,* and 21.1% (16/76) were *Se. barraudi*. Interestingly, the newly identified species, *Se.* sp1 and *Se.* sp2*,* were found exclusively in eastern Taiwan, and were collected from two (2.6%) and five (6.6%) sites, respectively. The *Ph. kiangsuensis* specimen was captured at only one locality in Taitung.

The number of sand flies captured is summarized in Table 4. The predominant species was *Se. iyengari* (*n* = 1210, 93.7%)*,* followed by *Se. barraudi* (*n* = 49, 3.8%) and *Se. squamipleuris* (*n* = 21, 1.6%). Only two (0.2%) and nine (0.7%) individuals of *Se.* sp1 and *Se.* sp2 were collected, respectively. Among all collected sand flies, only one *Ph. kiangsuensis* (*n* = 1, 0.1%) was captured in Taitung, despite two separate efforts in the same locality (Additional file 2: Table S1, Nos. 71 and 112). On average, the number of sand flies captured per light trap per night was 9.9 (287/29) in western Taiwan and 21.38 (1005/47) in eastern Taiwan (Table 4). Species richness was the highest in Taitung, particularly downstream of Butterfly Valley in Yan-Ping Township, where all six species of sand flies were collected (Table 5; Additional file 2: Table S1, Nos. 71 and 112). Generally, the richness (6) in eastern Taiwan was higher than in western Taiwan (3). The general low diversity (< 0.303) and evenness (< 0.65) index, and higher relative abundance of *Se. iyengari* (> 0.833) in each city, suggesting the dominant species of *Se. iyengari* in Taiwan. Although New Taipei and Pingtung have higher diversity and evenness, and lower relative abundance. The richness and abundance were two and four, which resulted in bias. Moreover, in Changhua, Chiayi, and Orchid Island, the *Se. iyengari* was the exclusive species we observed (Table 5).

**Table 4:** Sand fly species and their numbers collected in Taiwan during the 2017–2018 survey

Area	*Ph* *kiangsuensis*		*Se* *iyengari*		*Se* *barraudi*		*Se* *squamipleuris*		*Se.* sp1		*Se.* sp2		Total
	Female	Male	Female	Male	Female	Male	Female	Male	Female	Male	Female	Male	
Western^a^			243 (8.4)	23 (0.8)	13 (0.4)		8 (0.3)						287 (9.9)
Eastern^a^	1(0.02)		842 (17.9)	102 (2.12)	32 (0.7)	4 (0.09)	13 (0.3)		2 (0.04)		8 (0.2)	1 (0.02)	1005 (21.38)
Total (%)	1 (0.1%)	0 (0%)	1085 (84.0%)	125 (9.7%)	45 (3.5%)	4 (0.3%)	21 (1.6%)	0 (0%)	2 (0.2%)	0 (0%)	8 (0.6%)	1 (0.1%)	1292 (100%)

^a^The trap numbers set in western and eastern areas were 29 and 47, respectively. The number of sand flies per trap was listed in parentheses

**Table 5:** The relative abundance, richness, diversity, and evenness of sand fly species in each city of Taiwan during the 2017–2018 survey

District	Richness	Abundance	Relative abundance						Diversity	Evenness
			*Ph.* *kiangsuensis*	*Se.* *iyengari*	*Se.* *barraudi*	*Se.* *squamipleuris*	*Se.* sp1	*Se.* sp2		
Western (*n* = 29)										
Taipei (A)	2	59	0	0.864	0.136	0	0	0	0.238	0.573
New Taipei (F)	2	4	0	0.750	0.250	0	0	0	0.500	0.811
Taoyuan (H)	2	31	0	0.935	0	0.065	0	0	0.125	0.345
Hsinchu (J)	2	20	0	0.950	0	0.050	0	0	0.100	0.286
Taichung (B)	2	19	0	0.947	0	0.053	0	0	0.105	0.297
Nantou (M)	2	12	0	0.833	0	0.167	0	0	0.303	0.650
Changhua (N)	1	68	0	1	0	0	0	0	0	–
Chiayi (Q)	1	4	0	1	0	0	0	0	0	–
Tainan (D)	3	66	0	0.924	0.061	0.015	0	0	0.144	0.279
Pingtung (T)	2	4	0	0.750	0	0.250	0	0	0.500	0.811
Subtotal	3	287	0	0.920	0.045	0.028	0	0	0.139	0.287
Eastern (*n* = 47)										
Yilan (G)	3	24	0	0.875	0.083	0	0	0.042	0.236	0.415
Hualien (U)	4	337	0	0.979	0.015	0.003	0	0.003	0.041	0.085
Taitung (V)	6	615	0.002	0.917	0.047	0.020	0.003	0.011	0.156	0.212
Orchid Island (V)	1	29	0	1	0	0	0	0	0	–
Subtotal	6	1005	0.001	0.939	0.036	0.013	0.002	0.009	0.116	0.165

Diversity was calculated using Simpson’s dominance index (1-D); evenness was calculated by dividing the Shannon–Wiener *H*′ by ln(richness)

Tests were conducted to identify the blood meal in sand flies. Only one *Se. iyengari* collected from Taichung (Additional file 2: Table S1, No. 50) was found to be visibly blood-fed. This blood-engorged *Se. iyengari* and the suspected vector, *Ph. kiangsuensis*, were examined individually, whereas the other specimens were pooled for detection. Sequencing confirmed that the blood meal of the engorged *Se. iyengari* was *Homo sapiens* (Lane 1 of Additional file 1: Fig. S5B and Additional file 2: Table S1). However, other sand flies, including *Ph. kiangsuensis*, were negative for foreign blood (Additional file 1: Fig. S5). In addition, all samples were negative for DNA of *Leishmania* spp. (Additional file 1: Fig. S6).

## Discussion

In this study, we conducted a nationwide survey of phlebotomine sand flies in Taiwan. Molecular identification revealed four previously reported species (*Ph. kiangsuensis, Se. iyengari*, *Se. barraudi*, and *Se. squamipleuris*) and two that were reported for the first time. Species diversity in eastern Taiwan was higher than that in western Taiwan; all six species of sand fly were collected in Taitung of Taiwan. The predominant species was *Se. iyengari*, which was distributed across the entire island of Taiwan. We collected only one *Ph. kiangsuensis* specimen, a suspected vector of *Leishmania* spp. in Taitung. One engorged *Se. iyengari* specimen was found to be positive for the human gene. All samples were negative for *Leishmania* DNA, suggesting that the risk of *Leishmania* transmission in Taiwan is relatively low.

Large-scale surveillance of sand flies in Taiwan was previously conducted in 1970 and 1996. Previous studies documented eight species, namely *Ph. kiangsuensis*, *Se. iyengari*, *Se. barraudi*, and *Se. squamipleuris*, and four non-nominated sand flies that were morphologically distinct from the first four species; however, the characteristics of these non-nominated sand flies were not described [5, 12]. Lin et al. also surveyed the fauna of sand flies in Taiwan and detected six species: *Se. iyengari*, *Se. barraudi*, and *Se. squamipleuris*, plus three additional non-nominated sand flies. However, the descriptions of these three newly discovered sand flies were not provided [14]. In this study, we updated the sand fly species count in Taiwan to six species using molecular methods, including two species that were reported for the first time. Interestingly, the *Se. barraudi* from Taiwan and other Asian countries were separated into three distinct lineages (SG1, SG2, and Taiwan strain) in the phylogenetic analysis. The interspecific divergences between these three *Se. barraudi* strains are 0.1686–0.2354 in *COI*. These observations suggest that *Se. barraudi* in Taiwan is likely a member of *Se. barraudi* complex (*Se. barraudi* sensu lato [s.l.]) and may represent either a unique strain or a sibling species. Our analysis also supported the possible existence of cryptic species in previously reported *Se. barraudi* [32, 33]. However, a previous study proposed *Se. barraudi kwangsiensis* as a subspecies distributed in southern China [34]. Since no DNA barcode is currently available for *Se. barraudi kwangsiensis*, it remains unclear whether the population identified in Taiwan as *Se. barraudi* is conspecific with the subspecies from southern China. Further molecular comparison is needed to clarify their taxonomic relationship. In addition, distinguishing *Se. squamipleuris* from *Se. indica* has long been challenging due to their similar morphological characteristics, and some previous studies have even suggested they may represent the same species [35, 36]. In the present study, the phylogenetic tree analyses based on the sequences of *COI* and *Cytb* found that *Se. squamipleuris* from Taiwan, China, and Kenya clustered with *Se. indica*. The interspecific divergence in *COI* between these two species (0.0517) fell within the observed range of the intraspecific divergence of *COI* (0.0012–0.0584). In addition, the interspecific divergence of *Cytb* (0.0352) was below the commonly accepted 0.05 threshold. Therefore, our data supports that *Se. squamipleuris* and *Se. indica* is conspecific and probably the same species. For *Se. iyengari*, all the *COI* sequences for various countries formed a supergroup (bootstrap value = 100). However, the *Se. iyengari* from Taiwan further formed a monophyletic clade with a bootstrap of 99, and was separated from those from China, Thailand, Bangladesh, Bhutan, and Malaysia. The *COI* interspecific divergence of *Se. iyengari* between Taiwan and other Asia countries (0.0912) fell out of the intraspecific divergence (0.0012–0.0584). Our results suggested the *Se. iyengari* observed in Taiwan likely belongs to the *Se. iyengari* complex and should be referred to *Se. iyengari* s.l. This is consistent with Cates et al.’s study that the morphological feature of *Se. iyengari* in Taiwan is different from the reported *Se. iyengari*, and is probably a subspecies of *Se. iyengari taiwanensis* [12]. On the other hand, *Se. iyengari* was historically considered synonymous with *Se. hivernus* and *Se. khawi*, but was later recognized as a distinct species based on subtle morphological differences [12, 33, 37, 38]. The data from the phylogenetic tree and intra- and interspecific divergence analysis of this study support that *Se. iyengari* and *Se. khawi* are distinct species and agree with the previous study [32, 39]. However, the *Se. iyengari* (except the sample from Taiwan) and *Se. hivernus* formed a monophyletic clade, which is consistent with previous studies [32, 39]. Moreover, the low *COI* interspecific divergence (0.0416) was observed between these two species in this study. When taken together with the present and previous studies, we proposed that *Se. iyengari* (except the sample from Taiwan) and *Se. hivernus* are probably the same species. The phylogenetic tree of *COI* and *Cytb* also showed that *Se.* sp1 and *Se.* sp2 formed two monophyletic clades apart from other *Sergentomyia* sand flies (a single clade of *Se.* sp2 was also observed in the tree constructed by *18S rDNA*). The interspecific divergence of *Se.* sp1 and *Se.* sp2 from other species ranged from 0.1894 to 0.4563, supporting their distinction from other species. Also, the low sequence identity (ranging from 89.6 to 92.2%) to the species record in the NCBI database suggests that these two strains are probably unique species. However, because we only collected a few samples of these two species in this study, further investigation should be conducted, including their morphological features, for a more detailed understanding.

*Phlebotomus sergenti* is the principal vector for *L. tropica*. This species is primarily found in the Mediterranean area, Western Asia, and Southern Asia, where the climate has characteristics of relatively higher temperature and lower precipitation [40]. In Taiwan, *Ph. sergenti* has not been recorded in previous and present studies, possibly due to the geographical limitation and the high temperature in summer, usually accompanied by higher precipitation (https://climate.cwa.gov.tw/). *Ph. kiangsuensis* is a suspected vector of *Leishmania* spp. in Taiwan, with previous captures traced back to 1970 [12]. At that time, only three female specimens were collected from Taitung. In this study, we were unable to collect *Ph. kiangsuensis* from the same location where it was previously found, likely due to landscape alterations [14]. However, we identified one *Ph. kiangsuensis* specimen, confirmed by molecular sequencing, in another village of Taitung (Additional file 2: Table S1, No. 71). Despite returning to the same location, no additional *Ph. kiangsuensis* were captured (Additional file 2: Table S1, No. 112). Our findings indicate that *Ph. kiangsuensis* still exists in Taitung, Taiwan, but at a very low density. However, the finding of *Se. iyengari* as the predominant species in this study aligns with that from previous studies [12, 14]. In contrast to previous studies, which reported difficulties in collecting *Se. iyengari* in western Taiwan, except Nantou [12, 14], we successfully captured this species in several cities and counties in western Taiwan. This observation suggests that this species has a broader habitat range than previously anticipated. The richness and density of sand flies in eastern area were higher than those in western area. In Taiwan, the level of urbanization is higher in the western area than in the eastern area. Our observation is consistent with those from previous studies, suggesting that increased urbanization is negatively associated with sand fly density [41]. We also hypothesize that the construction of major transportation networks and industrial parks in western Taiwan, along with pollutant emissions, may have restricted sand fly distribution [42]. On the other hand, the diversity and evenness are low in eastern and western Taiwan probably due to the predominant role of *Se. iyengari*.

In this study, all sand flies tested negative for *Leishmania* spp. DNA, which suggests that the risk of leishmaniasis transmission in Taiwan is relatively low. This finding aligns with the fact that only six indigenous cases have been reported in Taiwan over the past six decades [5–7]. However, the vector responsible for the *Leishmania* spp. transmission in Taiwan remains unclear. The suspected vector, *Ph. kiangsuensis,* is present in Taitung, where no case of leishmaniasis has been reported. Meanwhile, the species collected in the administration district with indigenous cases were all *Sergentomyia* sand flies. Although there is increasing evidence that supports the role of *Se. iyengari*, *Se. barraudi*, *Se. squamipleuris*, and other *Sergentomyia* sand flies as potential vectors of *Leishmania* spp., most studies have only detected the presence of *Leishmania* DNA in sand flies, which does not confirm their ability to transmit the disease [22, 23, 43–45]. A previous investigation in a village with two indigenous cases of leishmaniasis found the epimastigotes in the *Se. iyengari* mid- and hindgut, although promastigotes were absent in both the foregut and salivary glands [5]. Therefore, the vector competence and capacity of *Sergentomyia* sand flies to transmit the *Leishmania* parasite must be evaluated in the future [20]. Furthermore, recent studies have suggested that fleas, ticks, and biting midges may also serve as potential vectors of the *Leishmania* spp. [46–50]. However, whether these arthropods play a significant role in the transmission of *Leishmania* in Taiwan remains to be elucidated and warrants further investigation.

Although *Sergentomyia* sand flies have been observed with traces of *Leishmania* spp. DNA, their preference for feeding on cold-blooded animals has been considered a limitation in their ability to transmit mammalian leishmaniasis [5, 51]. However, recent studies have detected mammalian blood, including human blood, in *Sergentomyia* sand flies, which suggests their potential role in *Leishmania* spp. transmission [45, 51]. In this study, we identified one *Se. iyengari* specimen engorged with human blood, which was consistent with previous findings and provided further evidence of interactions between *Se. iyengari* and humans [22]. However, since only a single specimen was identified, this may represent a rare event, and further investigation is needed to confirm the significance of such interactions.

In conclusion, we re-evaluated the taxonomy of sand fly fauna in Taiwan by molecular method and identified six species: *Ph. kiangsuensis*, *Se. iyengari* s.l., *Se. squamipleuris* (probably synonymous with *Se. indica*), and *Se. barraudi* s.l., which may represent a unique strain or sibling species. We also proposed two first-reported species, *Se.* sp1 and *Se.* sp2. These findings updated Taiwan’s sand fly record and provided new directions for taxonomic study. However, further investigations are needed to determine the vector responsible for *Leishmania* transmission in Taiwan. The extremely low density of *Ph. kiangsuensis*, and the absence of *Leishmania* spp. DNA in all specimens suggested the low risk of large-scale indigenous transmission. Nevertheless, with climate change and global warming, sand flies and leishmaniasis have expanded their geographical distribution. Continuous surveillance to monitor the sand fly incursion and domestic sand fly distribution is essential.

## Supplementary Information

Additional file 1.

Additional file 2.

## Data Availability

The datasets supporting the conclusions of this article are included within the article and in its additional file.

## Abbreviations

*18S rDNA*: 18S ribosomal DNA
*COI*: Cytochrome c oxidase subunit I
QGIS: Quantum Geographic Information System
NJ: Neighbor joining
ML: Maximum likelihood
*Cytb*: Cytochrome b
ITS: Internal transcribed spacer
*Ph.*: *Phlebotomus*
*Se.*: *Sergentomyia* [52]
