# Supplementary material for: A morphological and molecular study of phlebotomine sand flies of Taiwan reveals the record of six species
Source: Parasit Vectors. 2025 Oct 9;18:403. doi: 10.1186/s13071-025-07017-1 (PMC12512477; doi:10.1186/s13071-025-07017-1)
Supplement: Supplementary file 1 — Additional file 1. [file 13071_2025_7017_MOESM1_ESM.pptx]

## Slide 1
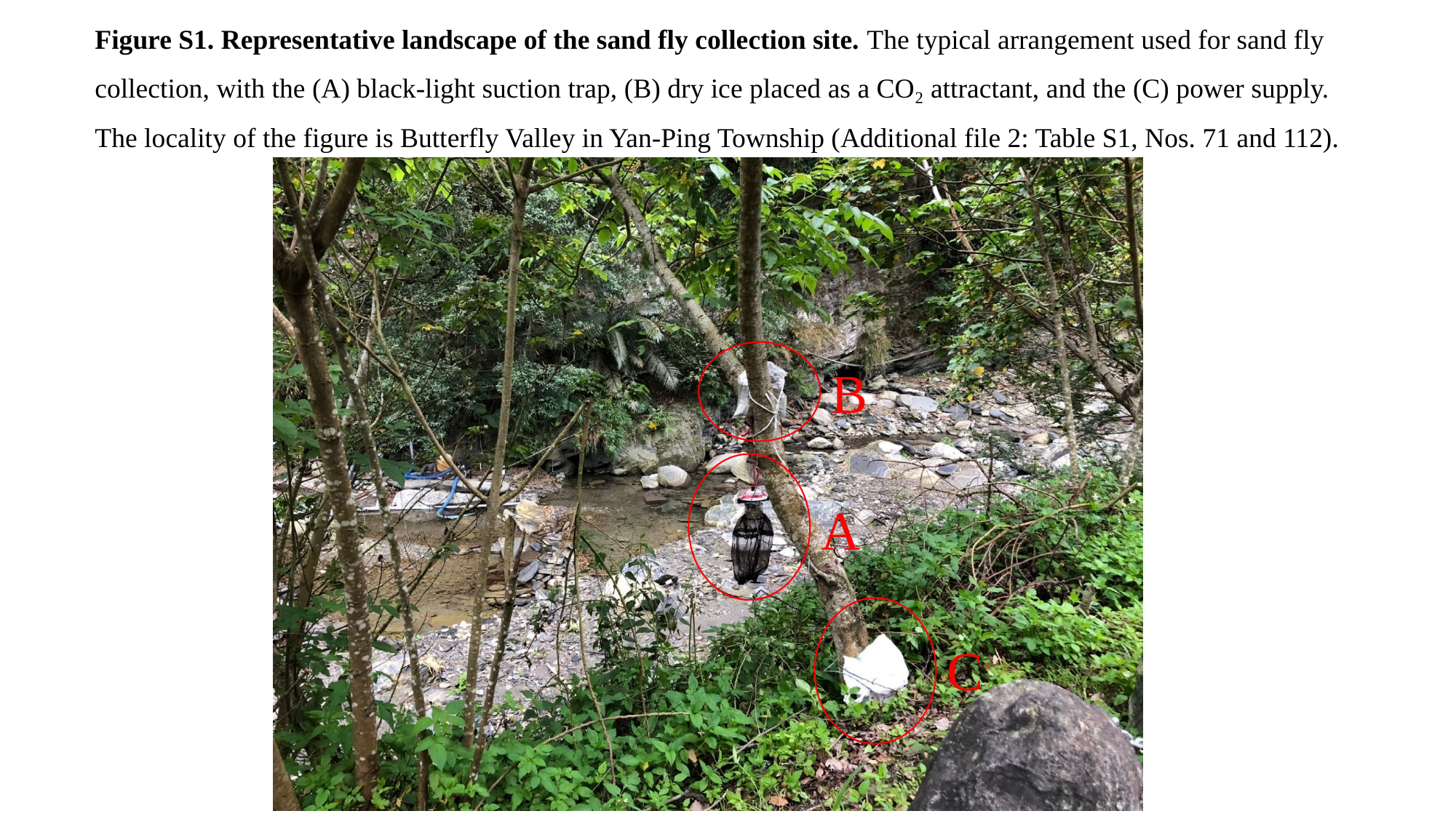

Figure S1. Representative landscape of the sand fly collection site. The typical arrangement used for sand fly collection, with the (A) black-light suction trap, (B) dry ice placed as a CO₂ attractant, and the (C) power supply. The locality of the figure is Butterfly Valley in Yan-Ping Township (Additional file 2: Table S1, Nos. 71 and 112).
B
A
C

## Slide 2
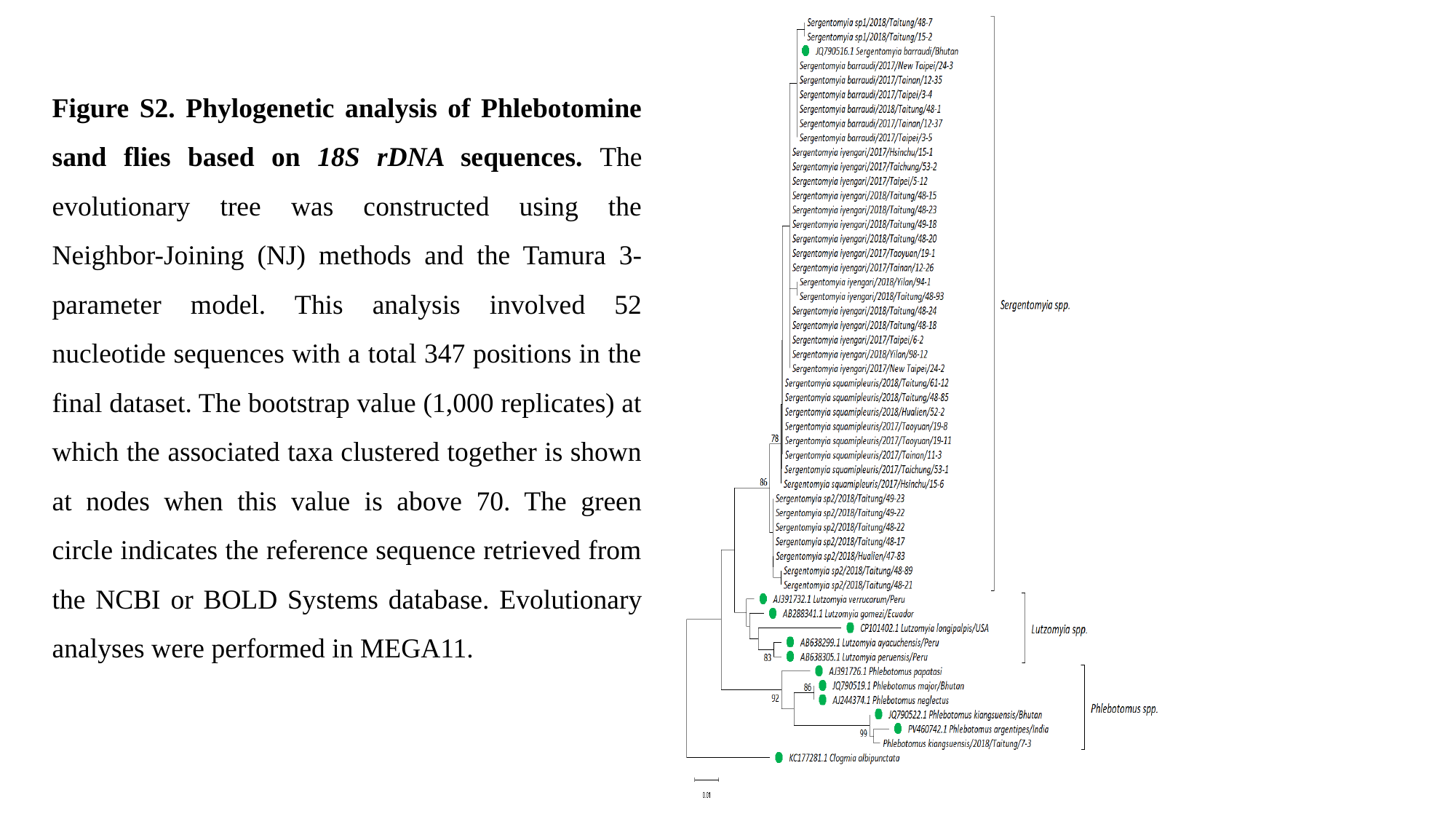

Figure S2. Phylogenetic analysis of Phlebotomine sand flies based on 18S rDNA sequences. The evolutionary tree was constructed using the Neighbor-Joining (NJ) methods and the Tamura 3-parameter model. This analysis involved 52 nucleotide sequences with a total 347 positions in the final dataset. The bootstrap value (1,000 replicates) at which the associated taxa clustered together is shown at nodes when this value is above 70. The green circle indicates the reference sequence retrieved from the NCBI or BOLD Systems database. Evolutionary analyses were performed in MEGA11.

## Slide 3
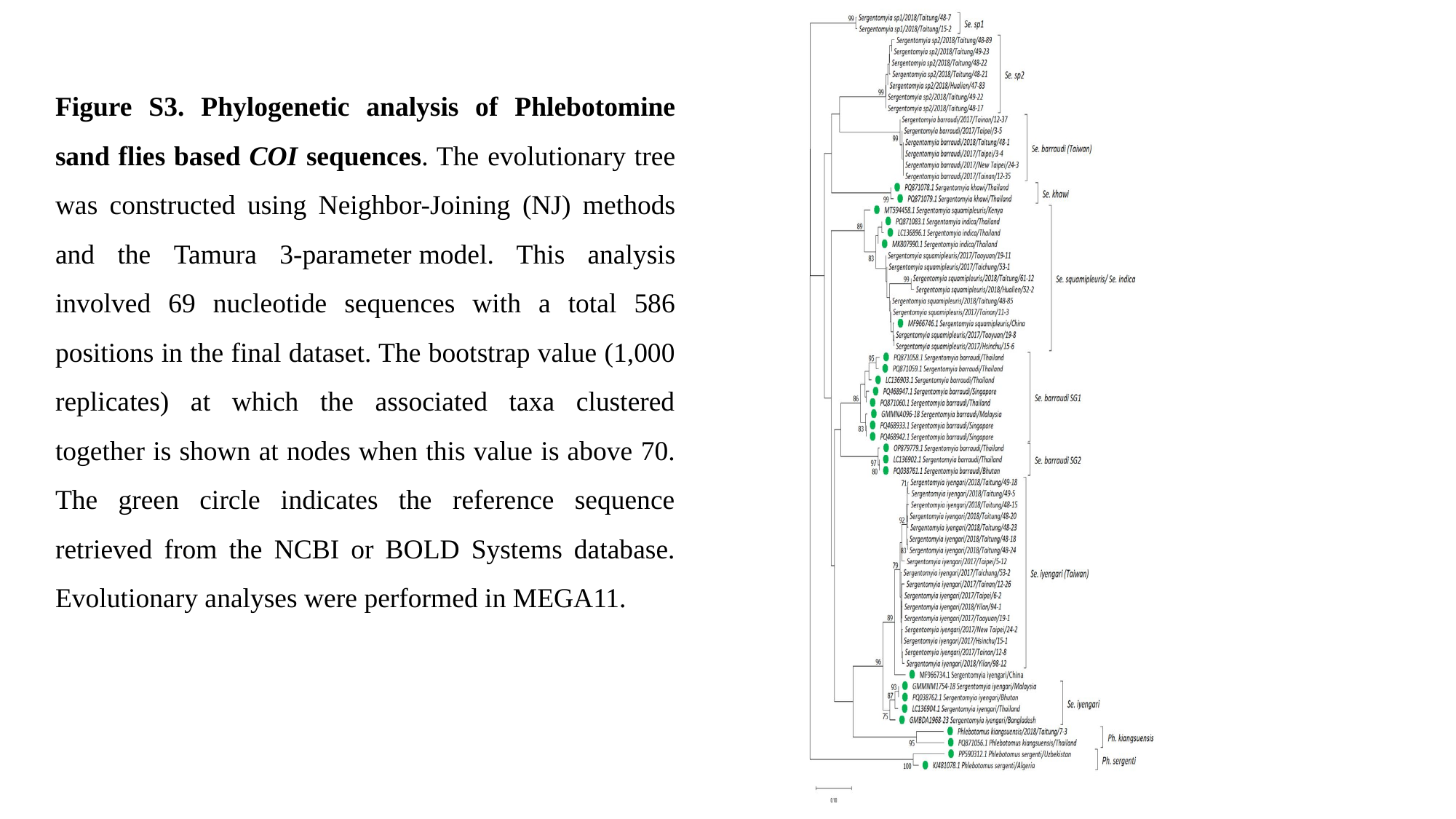

Figure S3. Phylogenetic analysis of Phlebotomine sand flies based COI sequences. The evolutionary tree was constructed using Neighbor-Joining (NJ) methods and the Tamura 3-parameter model. This analysis involved 69 nucleotide sequences with a total 586 positions in the final dataset. The bootstrap value (1,000 replicates) at which the associated taxa clustered together is shown at nodes when this value is above 70. The green circle indicates the reference sequence retrieved from the NCBI or BOLD Systems database. Evolutionary analyses were performed in MEGA11.

## Slide 4
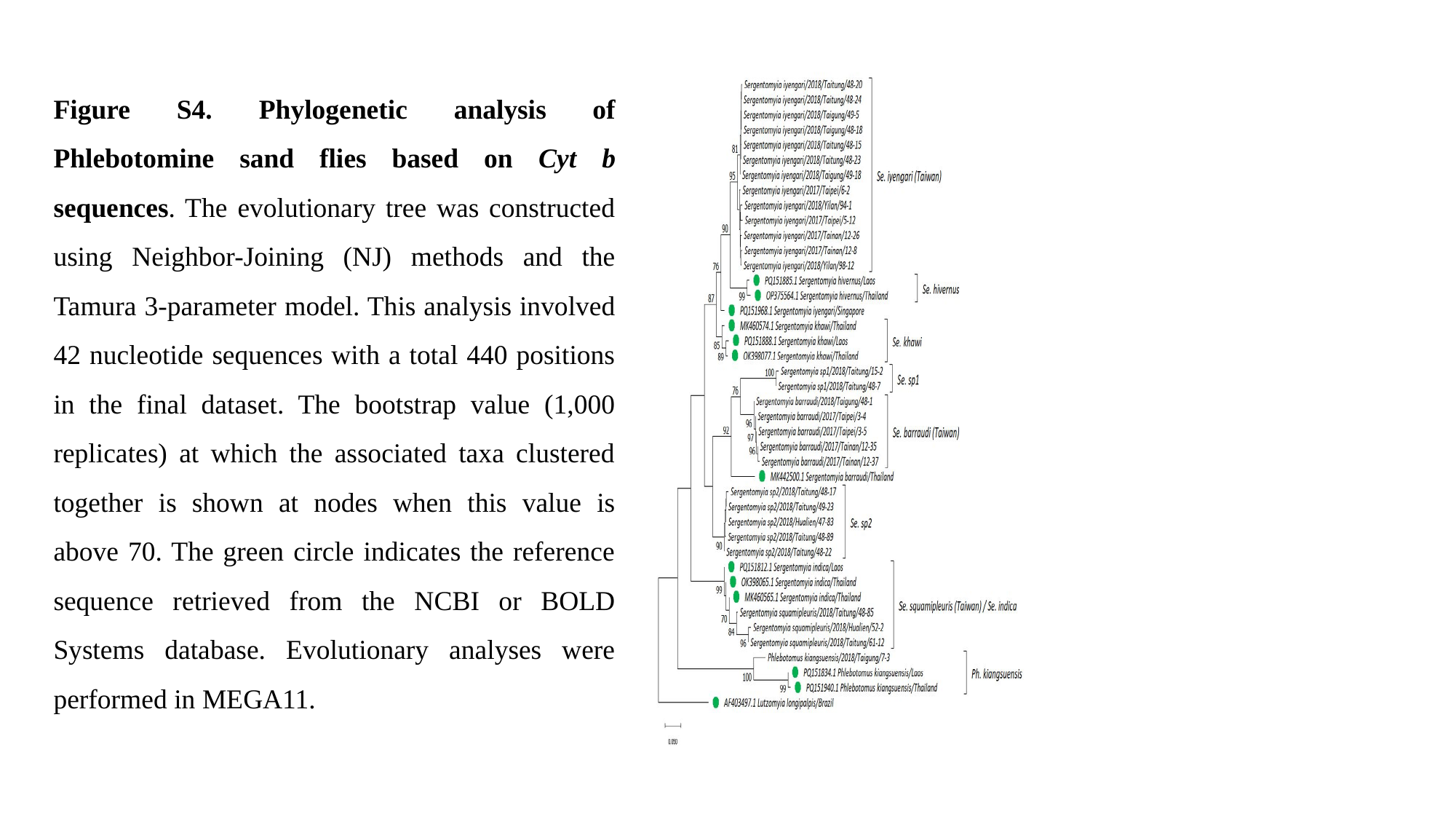

Figure S4. Phylogenetic analysis of Phlebotomine sand flies based on Cyt b sequences. The evolutionary tree was constructed using Neighbor-Joining (NJ) methods and the Tamura 3-parameter model. This analysis involved 42 nucleotide sequences with a total 440 positions in the final dataset. The bootstrap value (1,000 replicates) at which the associated taxa clustered together is shown at nodes when this value is above 70. The green circle indicates the reference sequence retrieved from the NCBI or BOLD Systems database. Evolutionary analyses were performed in MEGA11.

## Slide 5
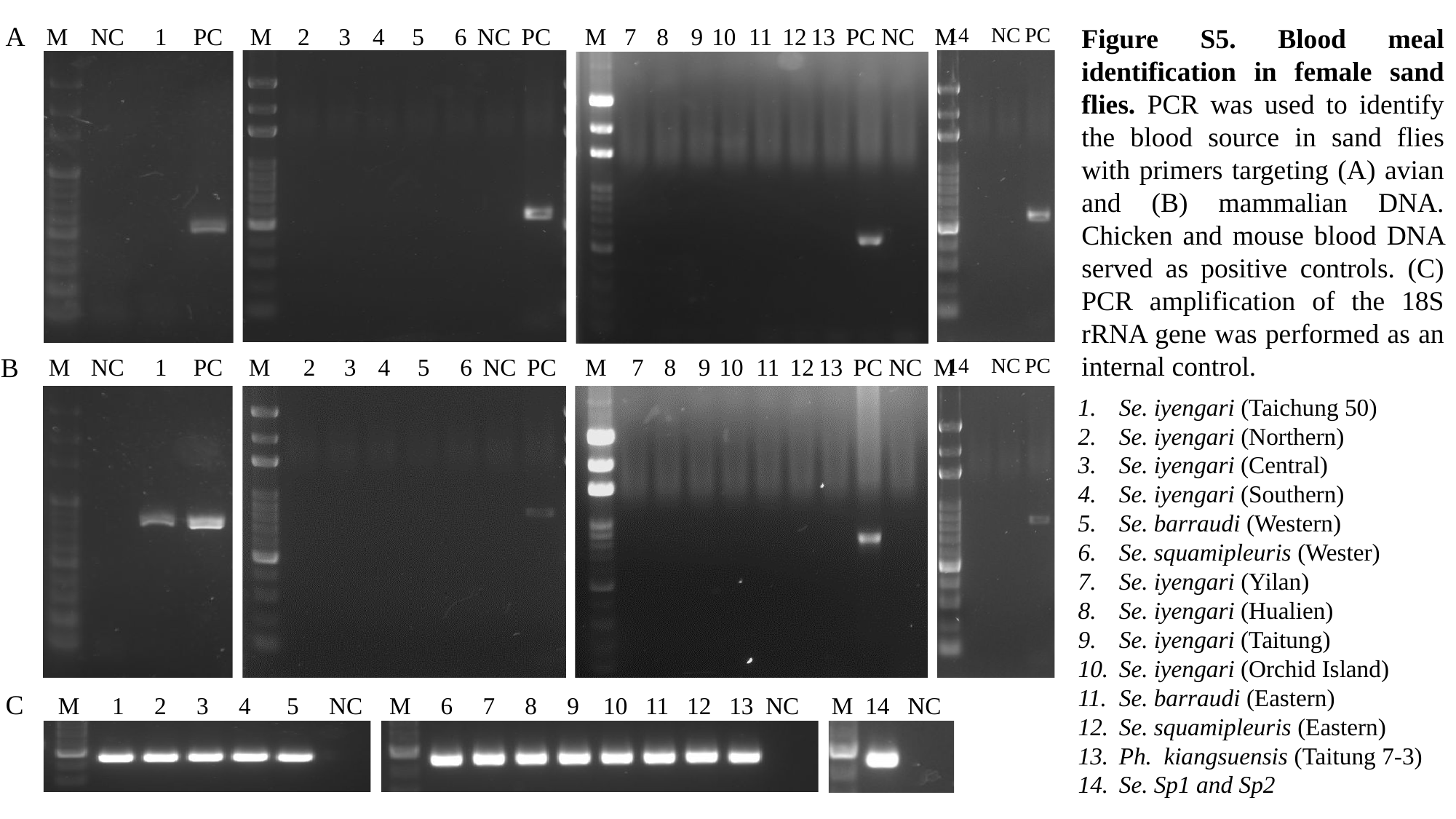

A
M
NC
1
PC
M
2
3
4
5
6
NC
PC
M
7
8
9
10
11
12
13
PC
 NC
M
14
 NC
PC
Figure S5. Blood meal identification in female sand flies. PCR was used to identify the blood source in sand flies with primers targeting (A) avian and (B) mammalian DNA. Chicken and mouse blood DNA served as positive controls. (C) PCR amplification of the 18S rRNA gene was performed as an internal control.
B
M
NC
1
PC
M
2
3
4
5
6
NC
PC
M
7
8
9
10
11
12
13
PC
 NC
M
14
 NC
PC
Se. iyengari (Taichung 50)
Se. iyengari (Northern)
Se. iyengari (Central)
Se. iyengari (Southern)
Se. barraudi (Western)
Se. squamipleuris (Wester)
Se. iyengari (Yilan)
Se. iyengari (Hualien)
Se. iyengari (Taitung)
Se. iyengari (Orchid Island)
Se. barraudi (Eastern)
Se. squamipleuris (Eastern)
Ph. kiangsuensis (Taitung 7-3)
Se. Sp1 and Sp2
C
M
1 2 3 4 5 NC 6 7 8 9 10 11 12 13 NC 14 NC
M
M
18SRNA
C

## Slide 6
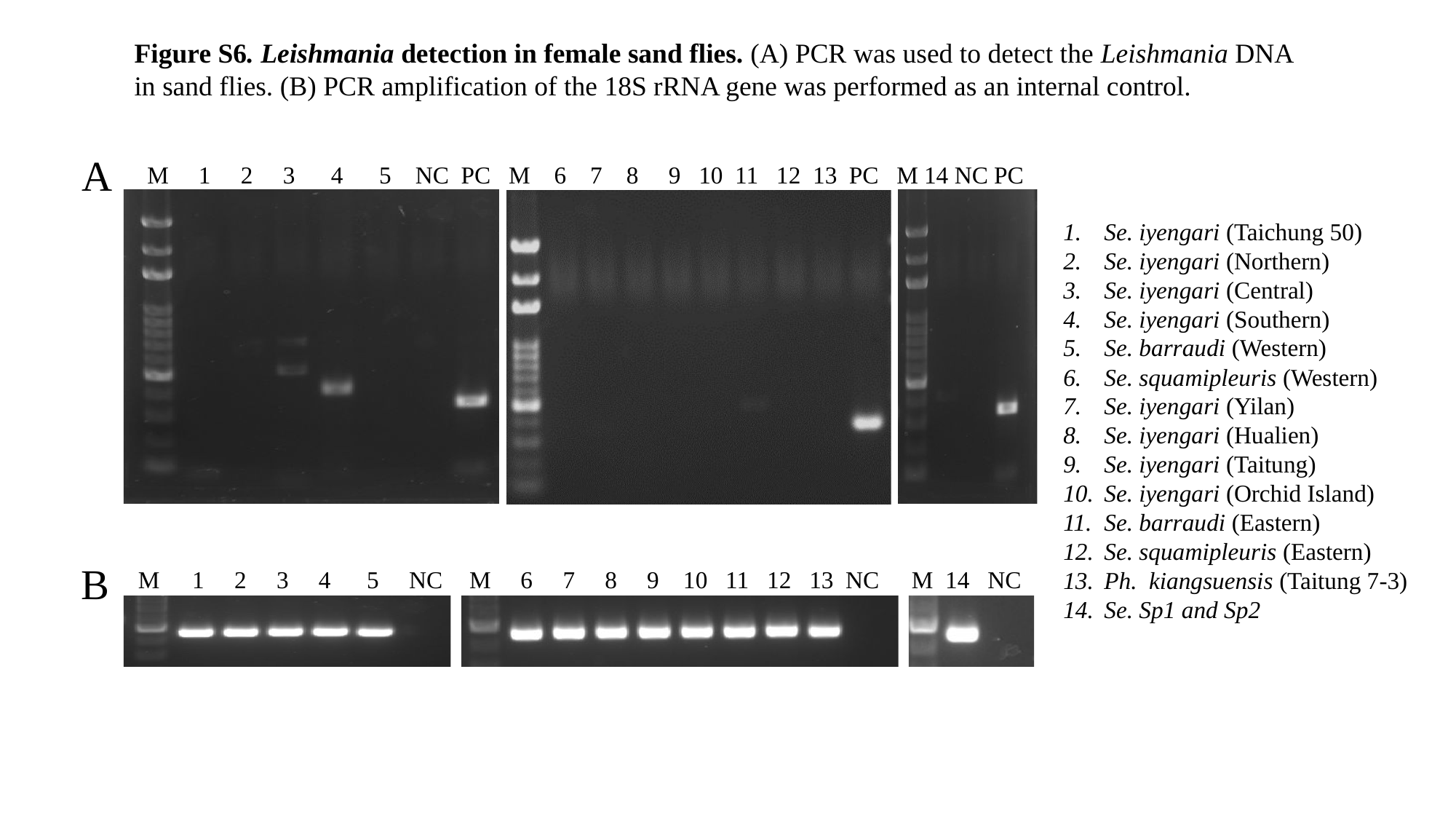

Figure S6. Leishmania detection in female sand flies. (A) PCR was used to detect the Leishmania DNA in sand flies. (B) PCR amplification of the 18S rRNA gene was performed as an internal control.
A
M 1 2 3 4 5 NC PC M 6 7 8 9 10 11 12 13 PC M 14 NC PC
Se. iyengari (Taichung 50)
Se. iyengari (Northern)
Se. iyengari (Central)
Se. iyengari (Southern)
Se. barraudi (Western)
Se. squamipleuris (Western)
Se. iyengari (Yilan)
Se. iyengari (Hualien)
Se. iyengari (Taitung)
Se. iyengari (Orchid Island)
Se. barraudi (Eastern)
Se. squamipleuris (Eastern)
Ph. kiangsuensis (Taitung 7-3)
Se. Sp1 and Sp2
B
M
1 2 3 4 5 NC 6 7 8 9 10 11 12 13 NC 14 NC
M
M
C
